# Growth differentiation factor-15 as a biomarker for intensive care unit-acquired weakness: a meta-analysis

**DOI:** 10.3389/fmed.2025.1486361

**Published:** 2025-01-30

**Authors:** Bing-Han Wang, Meng-Ying Qi, Zheng Yang, Gui-Lan He, Si-Ya Meng

**Affiliations:** ^1^Department of Critical Care Medicine, Huazhong University of Science and Technology Union Shenzhen Hospital/Shenzhen Nanshan People’s Hospital, Shenzhen, China; ^2^Department of Nursing, Huazhong University of Science and Technology Union Shenzhen Hospital/Shenzhen Nanshan People’s Hospital, Shenzhen, China

**Keywords:** growth differentiation factor-15, intensive care unit-acquired weakness, diagnostic utility, meta-analysis, review

## Abstract

**Background:**

Growth differentiation factor-15 (GDF-15) may be a potential biomarker for intensive care unit-acquired weakness (ICU-AW). In this study, we aimed to quantitative analysis the levels of GDF-15 in patients with ICU-AW and in non-ICU-AW, and then to determine its potential diagnostic utility.

**Methods:**

Two researchers separately conducted a systematic search of the relevant studies up to May 2023 in various literature databases (PubMed, Cochrane, Web of Science, Embase, and CINAHL). Studies were selected according to the inclusion and exclusion criteria, and quality evaluation of the included studies was conducted by using QUADAS-2 provided by Review Manager 5.3. The software packages Meta Disc (C1.4) and Stata17.0 were used for the meta-analysis. The data were combined with fixed-effects model, and the summary receiver operating characteristic curve was drawn to evaluate the overall diagnostic accuracy of GDF-15.

**Results:**

We identified 6 eligible studies comprising 401 patients with ICU-AW. The sensitivity, specificity, diagnostic odds ratio (DOR), and area under the curve (AUC) for the discriminative performance of GDF-15 as a diagnostic biomarker were 0.82 (95% confidence interval (CI):0.78–0.86), 0.83 (95% CI: 0.61–0.88), 21.39 (95% CI: 13.36–34.24), and 0.88 (95% CI: 0.85–0.91), respectively.

**Conclusion:**

GDF-15 is a candidate biomarker in diagnosing of ICU-AW from non-ICU-AW.

## 1 Introduction

Affecting approximately 50% of individuals who are critically ill, intensive care unit-acquired weakness (ICU-AW) is a common and serious consequence of critical illness (1, 2). ICU-AW can originate from critical illness myopathy, critical illness polyneuropathy, or critical illness neuromyopathy (3) and typically manifests as a symmetric, widespread weakness affecting the respiratory and limb muscles, but sparing the face and ocular muscles (4). Muscle tone is almost non-invasively reduced, and deep tendon reflexes may be diminished or normal (4). ICU-AW, also known as the syndrome of global weakness, is estimated to affect one million patients globally, including 75,000 patients in the United States (5). Severe functional impairment, extended mechanical ventilation, increased healthcare expenses, longer hospital stays, and higher mortality rates connected to the intensive care unit and hospitalization are all linked to ICU-AW (6, 7). The causes and mechanisms of ICU-AW remain poorly understood, resulting in an absence of targeted treatment alternatives for ICU-AW. Managing risk factors and hindering the initial advancement of the condition is essential, as the irregularities present in this phase may be reversible (8, 9). Early diagnosis necessitates early intervention; however, detecting ICU-AW in a timely manner is often difficult owing to states of unawareness in patients or the administration of sedatives, which can lead to delays in diagnosis and treatment (5, 10).

There is no “gold standard” diagnostic test for ICU-AW, even in the latest clinical practice guidelines (5). A diagnosis of ICU-AW can be accomplished through four approaches: using manual muscle testing with the Medical Research Council (MRC) score, conducting electrophysiological assessments (including electromyography and nerve conduction studies), performing muscle ultrasound, and examining muscle or nerve tissue pathology (11). However, these four methods have limited application in clinical practice. Manual muscle testing has considerable constraints as patients must be sufficiently alert and cooperative for the tests. Other diagnostic techniques—particularly muscle electrophysiological assessments and muscle ultrasound—are technically challenging and not commonly accessible in the ICU (12). In the past decade, serum biomarkers have been increasingly studied for early diagnosis of ICU-AW. Growth differentiation factor-15 (GDF-15) is one of the potential biomarkers that received great attention due to its close relationship with muscle wasting and decline in muscle mass.

GDF-15, first recognized as macrophage inhibitory cytokine-1 in 1997, is a stress-responsive component of the transforming growth factor-beta (TGF-*β*) cytokine superfamily (13). GDF-15 is predominantly expressed in myocardial cells, adipocytes, macrophages, endothelial cells, and vascular smooth muscle cells under pathological circumstances such as inflammation, insulin resistance, and oxidative stress (14). In addition, skeletal muscle cells produce GDF-15 in response to age-related stress, mitochondrial dysfunction, and mitochondrial proteotoxic stress, which may be essential in the progression of sarcopenia (15, 16). In healthy people, serum GDF-15 levels range from 200 to 1,200 pg./mL. Elevated GDF-15 levels upon ICU admission have been shown to be predictive of short-term and long-term mortality risk, particularly in patients with sepsis (17). Recently, multiple studies (18–23) have established a connection between GDF-15 and ICU-AW, and the association between elevated plasma GDF-15 levels and reduced expression of muscle microRNAs may explain the muscle atrophy observed in ICU-AW. Furthermore, the increased expression of GDF-15 in ICU patients may indicate macrophage activation and proinflammatory activities (20). Interest in utilizing GDF-15 as a biomarker for the diagnosis, prognosis, and/or risk stratification of patient populations suffering from ICU-AW is growing (24). However, the variability in findings across various studies is hindering efforts to determine the clinical significance of GDF-15 in these individuals. In this study, we collated original research articles referencing the role of GDF-15 in ICU-AW and performed a systematic evaluation to determine the value of GDF-15 as a diagnostic biomarker.

## 2 Materials and methods

### 2.1 Data sources and eligibility criteria

This meta-analysis was conducted in accordance with the Preferred Reporting Items for Systematic Reviews and Meta-Analyses (PRISMA) guidelines (25). All analyses were derived from studies that had been published previously. Consequently, obtaining ethical approval or patient consent was not applicable for this meta-analysis. Two independent reviewers conducted a selective literature search of several databases (PubMed, Cochrane, Web of Science, Embase, and CINAHL) during May 15–31, 2023. All pertinent articles were evaluated based on their titles and abstracts, and then reviewed for eligibility after being compiled and imported into NoteExpress software. MeSH terms or keywords used in the search were [(“Growth Differentiation Factor-15” OR “GDF-15” OR “Macrophage Inhibitory Cytokine-1” OR “MIC-1” OR “MIC1”) AND (“intensive care unit acquired weakness” OR “ICU-AW” OR “critical illness neuromuscular abnormality” OR “muscle weakness” OR “muscle atrophy” OR “muscle wasting and dysfunction” OR “critical illness polyneuropathy” OR “critical illness myopathy”)]. The references of the identified articles and associated reviews were searched manually to find any articles that might have been overlooked.

### 2.2 Study selection

**Figure 1:**
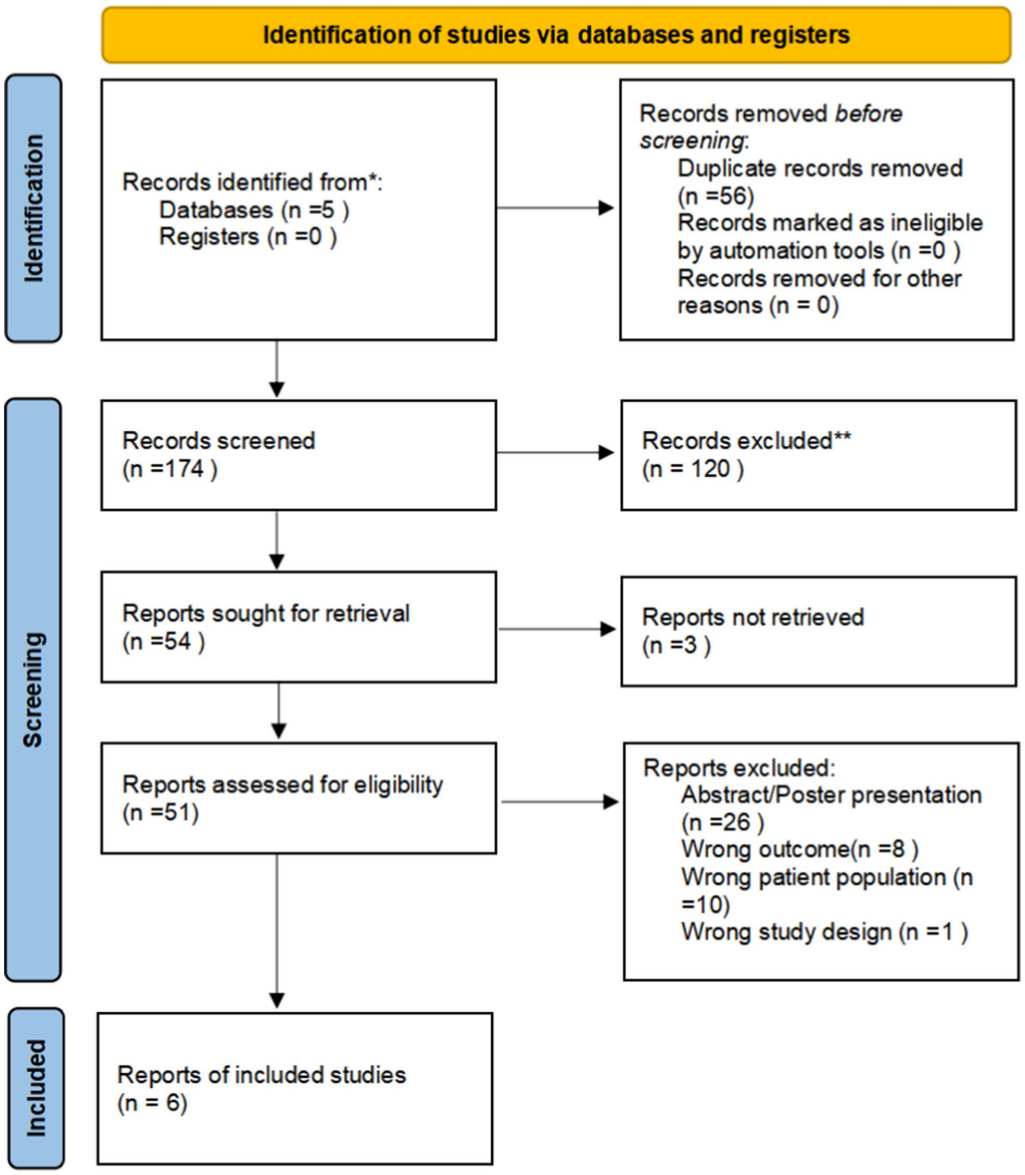
PRISMA 2020 flow diagram depicting the study selection procedure.

The selection process is shown schematically following the PRISMA 2020 flow diagram (Figure 1) (25). Studies in the full text were included if they satisfied the following criteria: (1) patient age ≥ 18 years; (2) proven diagnosis of ICU-AW by MRC score, electrophysiology, muscle ultrasound or muscle cell biopsy; (3) studies that documented the diagnostic characteristics of GDF-15 in ICU-AW; (4) adequate data for detailing or computing sensitivity and specificity. Exclusion criteria included: (1) studies lacking sufficient data and failed attempts to reach the authors; (2) duplicate studies; (3) studies with fewer than 10 cases; and (4) non-clinical research such as animal studies, reviews, conference abstracts, case reports, and meta-analyses.

### 2.3 Data collection

Data were extracted from articles by two independent reviewers. The collected data included the lead author’s name, publication year, country of the study population, sample type, number of patients, sensitivity, specificity, and cutoff value. In cases where sensitivity and specificity were not clearly stated in the article, these parameters were obtained from the area under the curve (AUC) using Engauge Digitizer software or manually computed from other available diagnostic accuracy metrics in the articles, or both. Attempts were made to contact authors of the original articles for the missing data, but these efforts were unsuccessful. When a study provided data for multiple comparisons, only one was selected for the final analysis based on its relevance to our research topic, concerns about heterogeneity, and to prevent a unit-of-analysis error. Any disagreements were settled through discussion or by reaching a consensus with a third reviewer.

### 2.4 Assessment of risk of bias

The quality and risk of bias of each study were examined using the revised Quality Assessment of Diagnostic Accuracy Studies-2 (QUADAS-2) criteria in Review Manager 5.3 software, based on specified criteria including patient selection, index test, reference standard, and flow and timing. All pertinent evidence was incorporated into the final analysis.

### 2.5 Data analysis

Meta-analysis was conducted using Meta Disc (C1.4) software. The primary outcomes included pooled sensitivity, specificity, positive likelihood ratio, negative likelihood ratio, and diagnostic odds ratio (DOR). Stata 17.0 was utilized to generate the summary receiver operating characteristic (SROC) curve and AUC along with their corresponding 95% confidence intervals (CIs). Cochrane’s *Q* and *I^2^* statistics were employed to confirm statistically significant heterogeneity among the studies, leading to the selection of a fixed-effects model. Owing to the inclusion of fewer than 10 studies, a funnel plot asymmetry analysis for assessing publication bias was not performed.

## 3 Results

### 3.1 Study selection and characteristics

Our literature search identified 261 potentially relevant studies (see Figure 1). These studies were reviewed based on titles, keywords, and abstracts, resulting in the exclusion of 210 studies due to reasons such as duplication, being outside the scope, or being basic or animal model studies. The remaining 51 studies were examined in detail, and six studies were included in the meta-analysis, which consisted of 401 patients with ICU-AW and 246 patients with non-ICU-AW based on our inclusion criteria. The features of the six included studies are summarized in Table 1. Among the six diagnostic studies, the participants comprised Chinese individuals (18, 22, 23), Americans (19), and British individuals (20, 21). All six studies were conducted prospectively. Only five of the studies reported cutoff values for GDF-15, which varied from 357.5 to 7,239 pg./mL (18–20, 22).

**Table 1:** Study characteristics of all the articles included in the diagnostic meta-analysis.

Authors	Year	Country	Design	Biomarker	Method	Sampling (n)	TP (n)	FP (n)	FN (n)	TN (n)	Cutoff (pg/ml)
Xie et al. (18)	2020	China	Prospective observational cohort study	GDF-15	Plasma; ELISA	95	47	13	3	32	1722
Rosenberg et al. (19)	2019	America	Prospective observational cohort study	GDF-15	Serum; ELISA	156	132	2	10	12	3,216
Bloch et al. (20)	2015	Britain	Prospective observational cohort study	GDF-15	Plasma; ELISA	27	8	1	12	6	7,239
Bloch et al. (21)	2013	Britain	Prospective observational cohort study	GDF-15	Plasma; ELISA	42	20	2	3	17	-
Deng et al. (22)	2022	China	prospective multicenter cross-sectional study	GDF-15	Serum; ELISA	235	76	14	41	104	357.5
Xie et al. (23)	2020	China	Prospective observational cohort study	GDF-15	Serum; ELISA	92	45	10	4	33	2,214

### 3.2 Risk of bias of included studies

**Figure 2:**
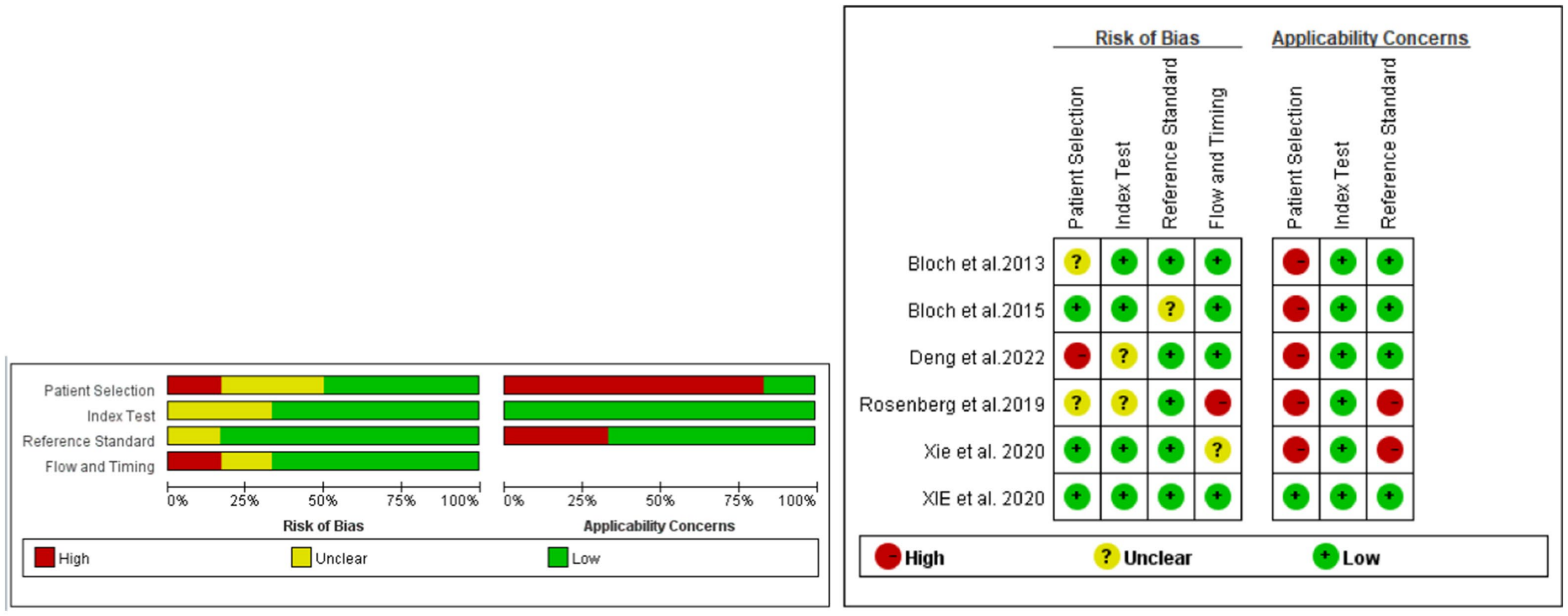
Evaluation of the risk of bias and applicability concerns of the included diagnostic studies using QUADAS-2.

The quality of the studies was assessed using QUADAS-2 (Figure 2). According to the QUADAS-2 evaluation, three of the six studies were at risk of bias in patient selection, two of the six studies had a risk of bias in the index test, one study showed a risk of bias in the reference standard, and two studies had a risk of bias of flow and timing. Despite these potential biases, all studies were included for further statistical analysis.

### 3.3 Synthesis of results

**Figure 3:**
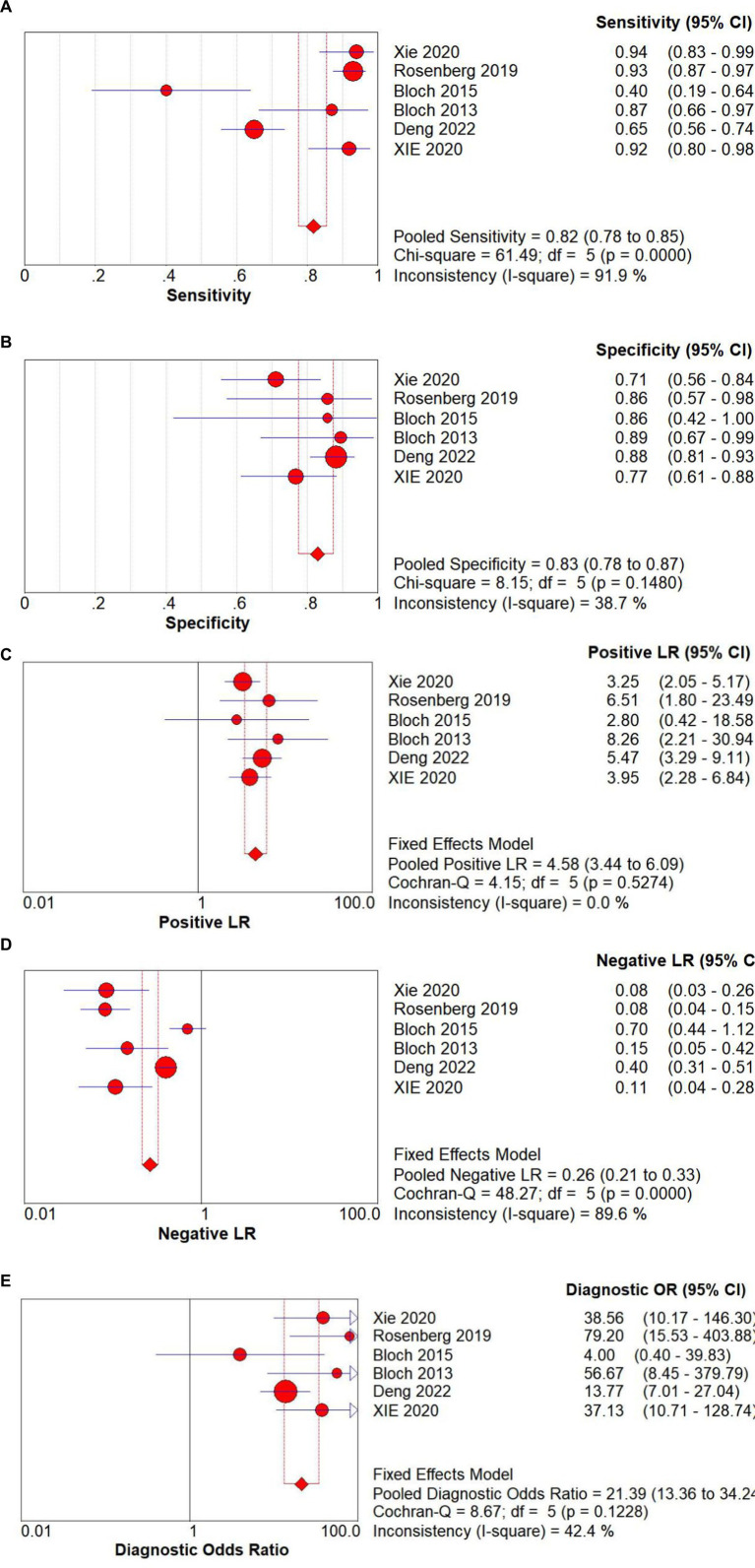
Forest plots of GDF-15 for the **(A)** sensitivity, **(B)** specificity, **(C)** positive LR, **(D)** negative LR, and **(E)** diagnostic odds ratio of the pooled data from the included studies.

**Figure 4:**
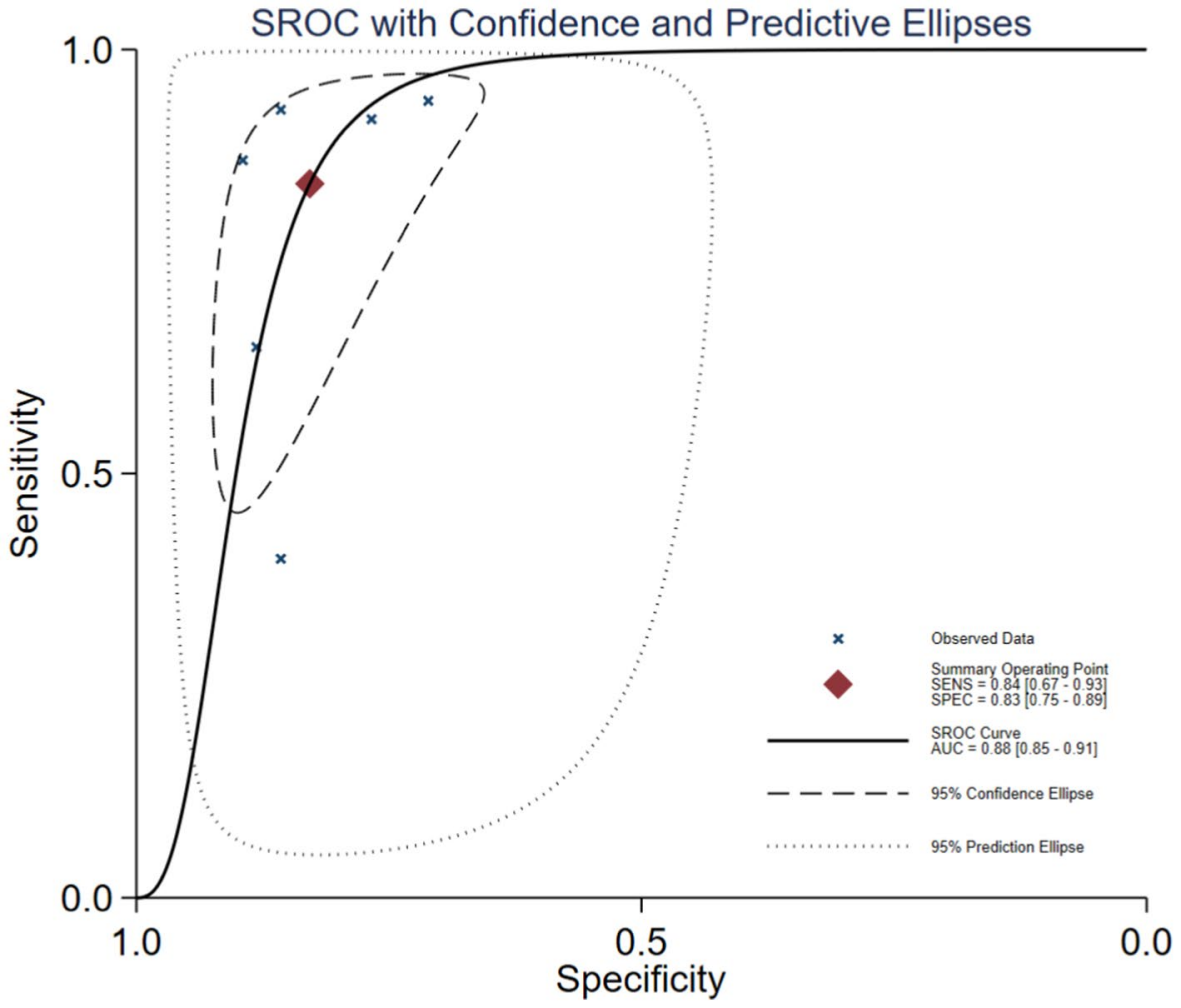
SROC curve of GDF-15 for the diagnosis of ICU-AW.

The Spearman correlation coefficient between the logarithm of sensitivity and the logarithm of (1 − specificity) was 0.714 (*p* = 0 111), indicating that there was no heterogeneity caused by threshold effects in this study (see Figure 3). A Cochrane-Q of DOR = 8.67 (df = 5, *p* = 0.1228) showed that there was no heterogeneity caused by non-threshold effects in this study. The diagnostic accuracy metrics for the included studies are presented in Table 2 and Figure 3. The overall pooled sensitivity, specificity, DOR, and AUC for GDF-15 in distinguishing ICU-AW from non-ICU-AW were 0.82 (95% CI: 0.78–0.86), 0.83 (95% CI: 0.61–0.88), 21.39 (95% CI: 13.36–34.24), and 0.88 (95% CI: 0.85–0.91), respectively. These metrics correspond to a positive likelihood ratio of 4.58 (95% CI: 3.44–6.09) and a negative likelihood ratio of 0.26 (95% CI: 0.21–0.33). The SROC curve is shown in Figure 4. These findings suggest that GDF-15 levels could be a valuable alternative biomarker for diagnosing ICU-AW when compared to non-ICU-AW.

**Table 2:** Measures of diagnostic accuracy in the selected studies.

Study	Sensitivity (95%CI)	Specificity (95%CI)	DOR (95%CI)
Xie et al. (18)	0.94 (0.84–0.99)	0.71 (0.56–0.84)	38.56 (10.17–146.30)
Rosenberg et al. (19)	0.93 (0.87–0.97)	0.86 (0.57–0.98)	79.20 (15.53–403.88)
Bloch et al. (20)	0.40 (0.19–0.64)	0.86 (0.42–0.99)	4.00 (0.40–39.83)
Bloch et al. (21)	0.87 (0.66–0.97)	0.90 (0.67–0.99)	56.67 (8.45–379.79)
Deng et al. (22)	0.65 (0.56–0.74)	0.88 (0.81–0.93)	13.77 (7.01–27.04)
Xie et al. (23)	0.92 (0.80–0.98)	0.77 (0.61–0.88)	37.13 (10.17–128.74)
Pooled data	0.82 (0.78–0.86)	0.83 (0.61–0.88)	21.39 (13.36–34.24)

95%CI, 95% confidence interval; DOR, diagnostic odds ratio; AUC, area under the curve.

## 4 Discussion

ICU-AW is a common complication in critically ill patients after prolonged stays in intensive care unit. ICU-AW can greatly impact the quality of life. Early recognization and diagnosis of ICU-AW can facilitate personalized treatments. Previous studies have reported that several biomarkers could be applied for early clinical diagnosis of ICU-AW. These biomarkers included lactate (26), neurofilament (27), urinary titin (28), MiR-181a (29), monocyte chemoattractant protein-1 (MCP-1) (30), glucose transporter type 4 (GLUT-4) (31), interleukin-6 (IL-6) (32), and growth differentiation factor-15 (GDF-15) (18–23).

Serum lactate is a valuable biomarker in critically ill individuals, but its connection to ICU-AW remains debatable (26). Plasma neurofilament level was proposed for early diagnosis of ICU-AW, but this biomarker is unable to distinguish between critical illness polyneuropathy and critical illness myopathy (33). Urinary titin levels can be an effective biomarker for diagnosing ICU-AW and are strongly linked to muscle atrophy (34), with increased urinary titin levels correlating with atrophy of the rectus femoris muscle but not with diaphragm thickness. In addition, urinary titin levels can vary by the timing of urine collection and various physiological factors (13). The increase in miR-181a shortly after ICU admission exhibited a high specificity (91%) for muscle atrophy within one week. However, its low sensitivity (56%) suggested that some patients at risk of muscle atrophy could be missed, thus this test cannot be relied upon for ruling out a diagnosis of acute muscle atrophy. It was reported that plasma MCP-1 level could be one of the risk factors for ICU-AW in patients with sepsis (29). However, studies with a larger sample size are required to confirm its diagnostic significance (30). Monitoring GLUT-4 provides some predictive value for ICU-AW in liver transplantation patients. An increased GLUT-4 level was associated with a low probability of ICU-AW, but the role in early diagnosis was limited (31). Animal studies have shown that IL-6 could contribute to increased muscle fatigue and reduced contractility of the diaphragm. Furthermore, recent clinical findings indicated a connection between IL-6 and the age-related reduction in muscle strength. This research highlighted the possible involvement of IL-6 in the formation of non-excitable muscle membranes during the early stages of critical illness, ultimately resulting in muscle weakness. The clinical implications of these findings need further investigation (32).

Recently, the clinical value of GDF-15 in the early diagnosis of ICU-AW is receiving increasing attention since GDF-15 is closely associated with muscle wasting and a decline in muscle mass. In our research, we identified six original research articles that assessed the potential of GDF-15 as a biomarker for differentiating ICU-AW from non-ICU-AW. Through a meta-analysis, we concluded that GDF-15 may be a reliable biomarker, with a pooled sensitivity of 82% and a specificity of 83%. In addition, there was no significant heterogeneity observed among the studies, suggesting that the findings were stable and reliable. Five of the six studies were encompassed within the 95% confidence interval of the SROC curve, with one study falling within the 95% prediction interval. The AUC value for the SROC curve is 0.88, indicating that GDF-15 exhibits very high diagnostic accuracy for ICU-AW.

Excessive catabolism represents a pivotal metabolic phenomenon in critically ill patients. The breakdown of muscle protein is a fundamental mechanism of catabolism, directly contributing to the development of ICU-AW (35). Degradation of muscle protein is believed to occur primarily through the ubiquitin–proteasome and autophagy–lysosome pathways. When the protein degradation pathway is aberrantly activated, protein degradation is accelerated, resulting in a reduction in muscle mass and muscle atrophy (36).

The cytokine GDF-15 is a principal regulator of the protein synthesis/catabolism balance and may be involved in the activation of the aforementioned proteolytic pathways (37). Abnormal expression of GDF-15 in the human body was found to result in a reduction in muscle protein synthesis, thereby contributing to the development of muscle atrophy. The MRC score for patients with ICU-AW decreased progressively over the course of their treatment, whereas plasma levels of GDF-15 showed a marked upward trend. By the seventh day of treatment, the GDF-15 levels in the ICU-AW group were considerably higher than those in the non-ICU-AW group (18). A study by Bloch et al. (20) demonstrated that GDF-15 may inhibit the expression of muscle microRNAs by enhancing the sensitivity of the TGF-*β* signaling pathway, thus contributing to muscle wasting. This conclusion resulted from observations on muscle biopsies from the rectus femoris muscle of patients with ICU-AW. Meanwhile, Xie et al. (23) observed that the loss of paraspinal muscle cross-sectional area and the rate of loss were significantly and positively associated with serum GDF-15 levels on the seventh day. This finding suggests that GDF-15, as a biomarker reflecting muscle wasting, has a strong intrinsic relationship with the objective measurement of paraspinal muscle mass through imaging. GDF-15 can be used in conjunction with the MRC score, which represents muscle function, to evaluate the degree of muscle wasting in patients. These parameters complement each other and have a certain correlation, particularly in cases where assessment of muscle strength through the MRC score is not feasible, such as with ICU patients under sedation or coma. Thus, as a biomarker, GDF-15 can assist in the timely diagnosis and assessment of patients with ICU-AW.

There are several limitations to this meta-analysis. First, the studies are confined to Chinese, American, and British populations. The small sample sizes with high selectivity of populations will require additional validation studies to determine the generalizability of our findings in clinically diagnosing ICU-AW. Second, we only included six articles after vigorous screening to select researches for meta-analysis, which might cause attrition bias with missed studies and participant drop-offs. Future prospective studies in a larger sample size and diverse patient populations are required to confirm our research findings here. In addition, before introducing GDF-15 into clinical practice, cutoff values for GDF-15 need to be established and validated internationally. In our analysis, cutoff values for GDF-15 for the difference between ICU-AW and non-ICU-AW or healthy controls were provided in five of the six studies. Consequently, large-scale prospective studies are required to identify a threshold targeted to different patient populations.

## 5 Conclusion

This meta-analysis suggests that GDF-15 could be a valuable biomarker for differentiating ICU-AW from non-ICU-AW. Clinicians may consider testing the GDF-15 level to early identify patients with ICU-AW. However, the small sample sizes of the studies included in the analysis indicate a requirement for further research with larger, well-designed prospective studies.

## Data Availability

The original contributions presented in the study are included in the article/supplementary material, further inquiries can be directed to the corresponding authors.
